# Co-infections of human herpesviruses (CMV, HHV-6, HHV-7 and EBV) in non-transplant acute leukemia patients undergoing chemotherapy

**DOI:** 10.1186/s12985-020-01302-4

**Published:** 2020-03-17

**Authors:** Imene Handous, Bechir Achour, Manel Marzouk, Sana Rouis, Olfa Hazgui, Ines Brini, Abderrahim Khelif, Naila Hannachi, Jalel Boukadida

**Affiliations:** 1 Laboratoire de Microbiologie, UR12SP34, Hôpital Farhat Hached, Université de Sousse, Faculté de Médecine de Sousse, Sousse, 4000 Tunisie; 2 Université de Sfax, Ecole Nationale d’Ingénieurs de Sfax, Sfax, 3038 Tunisie; 3 Service d’Hématologie, Hôpital Farhat Hached, Université de Sousse, Faculté de Médecine de Sousse, Sousse, 4000 Tunisie; 4 Université de Monastir, Faculté de Pharmacie de Monastir, Monastir, 5000 Tunisie

**Keywords:** Herpesviruses, Chemotherapy, Acute leukemia, Co-infection

## Abstract

**Background:**

Human herpesviruses (HHVs) remain latent after primary infection and can be reactivated in response to immunosuppression and chemotherapy. Little is known about their incidence, potential relationships, risk factors and clinical impact in non-transplant leukemia patients.

This study investigated prospectively incidence, risk factors, clinical impact and possible association of HHVs-(1–7) infections in patients with newly diagnosed acute leukemia.

**Methods:**

Study design involved longitudinal sampling before chemotherapy and in different phases of chemotherapy: post-induction, post-remission, and post-salvage during 2016–2018. A total of 734 plasma samples from 95 patients were analyzed by a qualitative, multiplex PCR for HHVs detection and a quantitative real-time PCR was used for cytomegalovirus (CMV) quantification. HHVs-(1–6) IgG and IgM antibodies were tested using immunoassays. Risk factors were analyzed by binary logistic regression and relationships between viruses were analyzed using the Chi-square or Fisher’s exact test as appropriate.

**Results:**

The overall seroprevalences of HHV-(1–6) IgG were high (> 80%). At least one herpes viral agent was detected in 60 patients (63.3%). CMV was the most commonly detected virus in the different phases of chemotherapy (19.4%), followed by HHV-6 (9.7%), HHV-7 (5.2%) and EBV (2.7%). HSV-1/2 and VZV DNA were not detected. Twenty-seven patients (28.4%) had more than one virus detected in the follow-up, with 23 who were co-infected. CMV/HHV-6 was the most frequent co-infection (69.5%, 16/23). HHV-6 infection (*p* = 0.008) was identified as a risk factor for CMV infection while salvage treatment (*p* = 0.04) and CMV infection (*p* = 0.007) were found to be independent risk factors for HHV-6 infection. CMV co-infection was associated with severe lymphopenia with an absolute lymphocyte count (ALC) (< 500/μL) (p = 0.009), rash (*p* = 0.011), pneumonia (*p* = 0.016) and opportunistic infections [bacteremia, *p* < 0.001 and invasive fungal infection, (*p* = 0.024)] more frequently than CMV mono-viral infections.

**Conclusions:**

Our data suggest that co-infection with HHVs, especially CMV and HHV-6, may contribute to the development of serious clinical manifestations with profound lymphopenia, pneumonia rash and increased risk for bacterial and fungal co-infections. These findings may suggest the synergistic effect of HHVs associated infection.

## Background

Until recently, viral infections in patients with hematological malignancies were concerns primarily the allogeneic hematopoietic stem cell transplant (allo-HSCT) recipients. The current large increase of intensive immunosuppressive chemotherapy regimens particularly in non-transplanted patients with hematological malignancies had a potential impact on increasing the incidence of viral infections in this group [1, 2].

After immunosuppression, viral infections may result from a new infection or from reactivation of latent infections. Viruses commonly involved are human herpesviruses (HHVs) that are divided into three groups: alpha (α), beta (β), and gamma (γ) HHVs. Herpes simplex virus 1,2 (HSV-1 and HSV-2) and varicella-zoster virus (VZV) are α-HHV, cytomegalovirus (CMV), human herpesvirus 6A,6B (HHV6-A and HHV-6B), and 7 (HHV-7) are β-HHVs while Epstein-Barr virus (EBV) and human herpesvirus 8 (HHV-8) are γ-HHVs [3].

CMV is one of the most important viruses of this family and is the subject of active research in allo-HSCT setting. Nevertheless, there is very little information about its incidence, its role and its impact in other hematologic settings, especially in leukemic patients. Similarly, in these settings, poor data is available concerning the other HHVs which can also lead to severe diseases. Most existing studies consist of retrospective analyses and case series whereas only limited information is available from prospective studies [2, 4–8]. The reported incidence range and outcomes of herpes viral infections among these patients groups are wide and variable due to a number of factors, including the study design, the type of disease, the intensity and duration of T-cell-mediated immune suppression [9].

Given their seriousness, considerable attention has been devoted to investigating factors that might increase their severity in allo-HSCT recipients. The major risk factors for CMV recurrence in non-transplant settings are the advanced disease, poor performance status and use of high dose steroids, fludarabine, alemtuzumab, bortezomib, and rituximab [2]. Interestingly, it was also described that co-infection between HHVs and in particular, CMV co-infection with other HHVs were important cofactors of lethal and severe diseases [10–12]. Nonetheless, the frequency or the impact of HHVs co-infections have been studied less extensively in acute leukemia patients undergoing chemotherapy.

This study aimed to evaluate prospectively the incidence of HHV infections in consecutive patients with acute leukemia at different stages of chemotherapy. The relationships among these viruses, their potential risk factors and especially the co-infection of CMV with other HHVs and its impact on clinical manifestations were analyzed.

## Materials and methods

### Study design and participants

This prospective study conducted between January 2016 and December 2018, included 95 patients with newly diagnosed acute leukemia, 52 acute lymphoblastic leukemia (ALL) (39 B-cell ALL and 13 T-cell ALL) and 43 acute myeloid leukemia (AML) who were diagnosed and treated at the Department of Hematology of Farhat Hached University Hospital. All patients were subject to a pretreatment assessment, including a complete history and physical examination, as well as routine baseline investigations required for diagnosis and staging according to each disease category by referring to the WHO criteria. Initial treatment regimens included for almost all patients two-chemotherapy phases: induction and post-remission therapy. After induction therapy, eligible patients with high-risk disease and a matched donor may go on to allo-HSCT while all others were assigned to post-remission therapy (consolidation and maintenance). Complete remission (CR) was retained when blasts cells percentage was less than 5% in the bone marrow samples without evidence of circulating blasts or extramedullary disease and with the recovery of peripheral counts. On the basis of their responses to induction therapy patients were distributed into two groups; patients with CR and patients with treatment failure (chemoresistance) when evaluation did not meet the criteria of CR. Salvage treatments were selected in case of primary refractory when bone marrow still had more than 5% of blasts at the end of the first or the second identical induction or at relapsed acute leukemia when the recurrence of disease is after CR.

The exclusion criteria included: preexisting cancer; hematological, or immunological diseases, and chronic viral infections (human immunodeficiency virus, hepatitis B or C). Patients, who received allogeneic or autologous hematopoietic stem cell transplantation, were excluded from this study upon beginning preconditioning for transplantation. Patients enrolled in this study did not receive any CMV specific antiviral therapy.

Our study design involved prospective sampling at the time of diagnosis (before chemotherapy) and in the different phases of chemotherapy, post-induction, post-remission and post-salvage chemotherapy. Follow-up samples were collected at a later time in the same period with the aim to properly evaluate the occurrence of herpes viral infections. For some patients, the number of follow-ups was limited due to early death or loss to follow-up samples. The median follow-up duration was 7.4 months range (1–36) from the start of chemotherapy. There are 734 blood specimens, (3–20) samples per patient with a median of seven samples per patient from which all plasma samples were isolated and stored at − 80 °C until DNA extraction. The study was approved by the Ethics Committee and Medical Research of Farhat Hached University Hospital of Sousse, ref: IRB registration number assigned by OHRP: IRB00008931 and informed consent was obtained from all participants enrolled in the study.

### Herpesviruses serology

Serum samples from all 95 patients before starting chemotherapy were tested by commercially available diagnostic kits. Enzyme-linked immunosorbent assay (ELISA) testing kit (Euroimmun, Germany) was used to test HSV-1/2 and VZV specific antibodies (IgG and IgM). Epstein-Barr nuclear antigen-1 (EBNA-1) EBNA-1 IgG and viral capsid antigen VCA EBV IgM, CMV IgG and IgM were analyzed with chemiluminescence assays by the Abbott Architect *i*2000SR and CMV IgG avidity by a commercial kit (Euroimmun, Germany) to assess whether or not the infection was primo-infection. Immunofluorescence assay kits (DiaSorin, Italy) were used to test the HHV-6 IgG and HHV-6 IgM antibodies. The interpretation of the serological tests followed the manufacturer’s instructions.

### Herpesviruses multiplex PCR detection

DNA was extracted from a 200-μL of plasma using a QIAamp DNA Mini Kit (QIAGEN, Germany), according to the manufacturer’s instructions and DNA was eluted in a final volume of 50 μL. Multiplex PCR was performed as described previously by Tanaka et al to amplify HHVs DNA (HSV-1/2, VZV, EBV, CMV, HHV-6A/B, and HHV-7) [13]. An electropherogram of the multiplex PCR products showed the obtained amplified fragments of the expected size when using the primer sets specific to HHVs products (Table 1 and Additional file 1**: Figure S1**). Randomly selected amplicons for the studied HHVs were then sequenced by the Sanger method, to confirm the identity of the PCR products of each virus in clinical samples. Both forward and reverse DNA strands were sequenced with the same specific primers using an ABI PRISM Big Dye Terminator Cycle Sequencing Reaction kit (version 3.1) on an ABI PRISM 3100 DNA sequencer (Applied Biosystems, Germany). HHVs were identified based on sequence alignment of amplicons at the NCBI web site confirming their viral identity.

**Table 1:** Primers used for multiplex PCR of human herpesviruses in the study

Viruses (GenBank accession)	Primer	Primer sequence (5′-3′)	Position	Product length (bp)
HSV-1/2 (HSV-1:M10792, HSV-2: M16321)	HSV-F	ATCCAGTACGTCTTTGTGGAGCCCAAG	HSV-1:3389	292
			HSV-2:3058	
	HSV-R	*TGAGGACAAAGTCCTGGATGTCCCTCT*	HSV-1:3680	
			HSV-2:3349	
VZV (X04370)	VZV-F	*TCCGACATGCAGTCAATTTCAACGTC*	49651	161
	VZV-R	*GGTCGGGTAGACGCTACCACTCGTTT*	49811	
EBV (NC_007605)	EBV-F	*CTTAGAATGGTGGCCGGGCTGTAAAAT*	153240	229
	EBV-R	ATCCAGTACGTCTTTGTGGAGCCCAAG	153468	
CMV (NC_001347)	CMV-F	GCGCGTACCGTTGAAAGAAAAGCATAA	80362	131
	CMV-R	TGGGCACTCGGGTCTTCATCTCTTTAC	80492	
HHV6A/B (HHV6A: NC_001664,HHV6B: AB021506)	HHV6-F	ATGCGCCATCATAATGCTCGGATACA	HHV-6A: 57837	183
			HHV-6B: 58791	
	HHV6-R	CCCTGCATTCTTACGGAAGCAAAACG	HHV-6A: 58019	
			HHV-6B: 58973	
HHV7 (NC_001716)	HHV7-F	GCCCGTTTTCGGAAATATTGGAGAGAT	55671	347
	HHV7-R	ACGCACGAGACGCACTTTTCTTAAACA	56017	

Primers and targets used in the Multiplex PCR assay for the simultaneous detection of herpesviruses (HSV-1/2, VZV, EBV, CMV, HHV-6A/B, and HHV-7). Each primer pair was designed with different amplicon size to identify each product with agarose gel electrophoresis

### CMV viral load

CMV viral load was performed in the first positive plasma samples (multiplex PCR) using *artus CMV RG PCR* kit (Qiagen, Germany) and Rotor-Gene Q (RGQ) instrument, according to the manufacturer’s recommendations. Results were recorded in copies/mL. The lower limit of detection for this assay is 57.1 copies/mL. High-level CMV DNAemia was defined in this study, as having quantitative CMV DNA in plasma ≥1000 copies/mL.

### Definition of infection

In this paper, definitions of viral infections were those according to published criteria [14] for CMV viral infection. CMV DNAemia was defined as the detection of viral DNA in samples of plasma. The same rationale was used to define EBV, HHV-6 and HHV-7 infection. If viral DNAemia resolved then became positive at a later time point this was considered as recurrent infection and was recorded as another detection.

Patients with more than one virus detected at one or more time points were classified as DNAemia caused by multiple viruses. Mono-viral infection was defined as the presence of DNA of one HHV detected at one or more time-point. A viral co-infection was assigned as the detection of DNA of more than one type of HHVs in the same sample. Dual and triple viral infections were defined as the presence of DNA of two and three different types of viruses, accordingly in the same sample.

We evaluated the hospital records of the patients and the daily clinical features for each day in which we tested a blood sample and investigated whether the patient had fever defined as ≥38.5 °C or > 38.0 °C that persisted for one hour, severe neutropenia with an absolute neutrophil count (ANC) of ≤500 cells/ μL; and prolonged neutropenia was defined if it persisted over 7 days**;** severe lymphopenia: with an absolute lymphocyte count (ALC) < 500/μL; thrombocytopenia: platelet count < 100 10 ^3^ / μL, anemia: hemoglobin < 11 g/L; skin rash, mucositis, conjunctivitis, pneumonia, central nervous system disorders (CNS), gastroenteritis, hepatitis and the presence of other opportunistic infections (bacteremia or invasive fungal) infections as shown by clinical examination, radiological, and microbiological criteria.

### Statistical analysis

The representation of patient’s data and detected viral infections were analyzed using the SPSS **20.0** statistical package. The number of infections was expressed as the prevalence within a given phase of therapy and it is a simple fraction of the number of patients experiencing at least one given infection compared to all patients undertaking the same phase of therapy. The Venn diagram was constructed by using a free online tool (http://bioinformatics.psb.ugent.be/webtools/Venn/). The prevalence of infections between different treatment phases and potential interaction was examined between EBV, CMV, HHV-6 and HHV-7.

Viral DNA findings were modeled as dichotomous variables and coded as positive at any level, that is to say no limit in the copy number exists for the distinction between a viral acute infection and latency. Thus, we labeled these viral DNA findings as infections, regardless of copy number. Univariate logistic regression analysis of risk factors for HHVs infections was performed, showing the association between viral infections and patient’s demographic and clinical characteristics using the Chi-square (X^2^) test or Fisher’s exact test as appropriate. Associations with significance level with *p-value <* 0.1 in univariate analysis were subsequently included in binary logistic regression models. The statistically significant independent association was considered if *p*-value *<* 0.05 after multivariate analysis. We divided viral infections into two groups based on the number of viruses detected: active mono-viral infections and co-infections. Due to the high level of CMV co-infection, we fit a model with combined covariate to access the joint and separate effects of CMV and other HHVs. This variable for CMV and other HHVs infections had three levels (1) active mono-viral infection with only CMV and no other HHVs infection (2) active co-infection with CMV and other HHVs (3) no active CMV infection.

## Results

### Patients’ characteristics

**Fig. 1:**
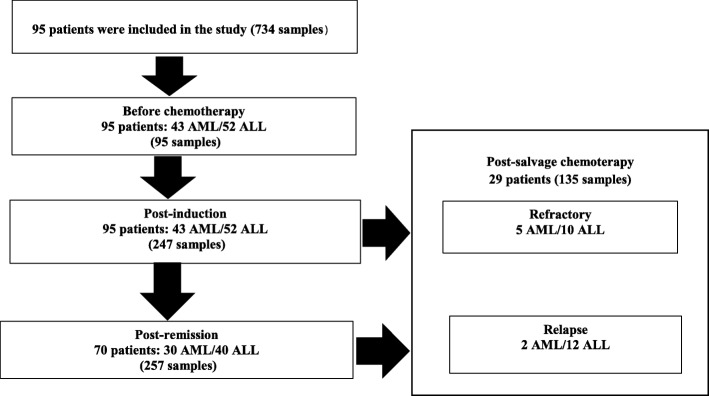
Patients’ flow chart depicting the process of enrollment for analysis. Acute leukemia patients were enrolled in the study at different phases of chemotherapy: before chemotherapy, post-induction, post-remission and post-salvage

Ninety-five newly diagnosed acute leukemia patients aged 1–64 years (mean 16) at the time of diagnosis were prospectively enrolled in the follow-up study before starting induction therapy. Patients’ characteristics are presented in Fig. 1**.** The male/female sex ratio was 1.96. The median follow-up duration was 4.8 months range (1–22.5) for patients with AML and 12.73 months range (1.1–36) for ALL. Seventy patients out of 95 had clinical and laboratory evidence of first remission after induction therapy, while 15 had primary refractory disease and 10 died (8 AML and 2 ALL) in aplasia. Fifty-four out of 70 patients were on continuous CR after post-remission therapy, 14 were in first relapsed and 2 AML patients died. Twenty-nine patients with relapsed or refractory acute leukemia were treated with salvage regimens. Eighteen out of 29 patients achieved CR following salvage therapy, whereas 6 patients were refractory and 5 ALL patients died at the end of the study. Overall we evaluated 734 plasma tests: 95 (from 95 patients), 247 (from 95 patients), 257 (from 70 patients), and 135 (from 29 patients) before chemotherapy, post-induction, post-remission, post-salvage, respectively.

### Herpesviruses serology

The overall IgG prevalences were 95% (91/95), 84,2% (80/95), 81% (77/95), 94,7% (90/95) and 97,9% (93/95) for HSV1/2, VZV, EBV, CMV and HHV-6 respectively, while the corresponding IgM prevalences were 3% (3/95),3% (3/95), 4% (4/95),5% (5/95) and 2% (2/95) respectively. The avidity of IgG antibodies against CMV was high in all these patients indicating past exposure.

### Analysis of single and multiple herpesviruses infections

**Fig. 2:**
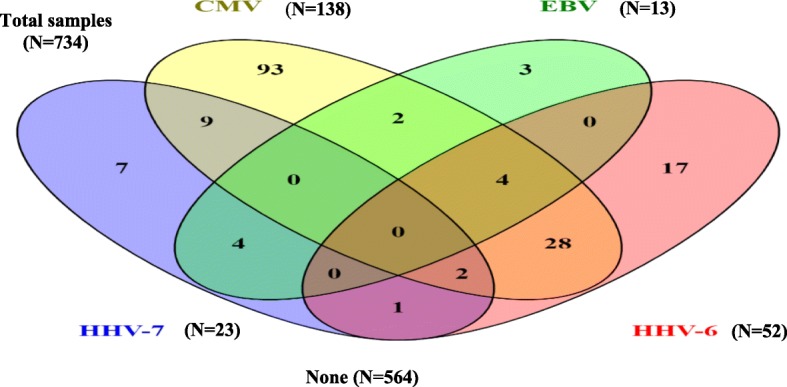
Single infections and co-infections detected in acute leukemia patients. Venn diagram shows the number of positive samples for each HHVs. Numbers of single infections can be seen at the ends of the diagram (*N* = 120), while co-infections can be seen at overlapping areas (*N* = 50)

Of the 95 patients, 60 were tested positive in at least one of their samples to at least one of the HHVs analyzed; 33 (34.7%) patients were single infected and 27 (28.4%) were multiply infected; 21 of these with two viruses and 6 with three viruses. Of 734 samples, 170 samples were positive for one or more viruses. Among all positive viral detections, 120 samples were single positive viral detection of which 93 were positive to CMV, 17 to HHV-6, 7 to HHV-7 and 3 to EBV. A total of 50 samples showed positive viral detection to more than one HHVs; 44 were dual infections and 6 were triple infections (Fig. 2**).** None of the samples examined were HSV-1, 2 and VZV positive. Neither patients at diagnosis with positive HHVs IgM nor patients with negative HHVs serology developed HHVs active infection during the study. There was no significant difference between AML and ALL groups regarding the presence of HHVs **(**Table 2**).**

**Table 2:** Distribution of herpesviruses in leukemic patients (ALL or AML)

Viral infection	Patients ***N*** = 95 (%)	Patients with AML ***N*** = 43 (%)	Patients with ALL ***N*** = 52 (%)	***P***
**Single CMV**	21 (22.1)	12 (27.9)	9 (17.3)	0.227
**Single EBV**	3 (3.1)	–	3 (5.8)	0.248
**Single HHV-6**	5 (5.3)	2 (4.6)	3 (5.8)	1.000
**Single HHV-7**	4 (4.2)	–	4 (7.7)	1.000
**CMV + HHV-6**	15 (15.8)	5 (11.6)	10 (19.2)	0.401
**CMV + HHV-7**	5 (5.3)	4 (9.3)	1 (2)	0.172
**EBV + HHV-7**	1(1.1)	–	1 (2)	1.000
**CMV + HHV6 + HHV7**	2(2.1)	2 (4.6)	–	0.202
**CMV + EBV + HHV6**	2(2.1)	1 (2.3)	1 (2)	1.000
**EBV + HHV6 + HHV7**	1(1.1)	1 (2.3)	–	0.452
**EBV + CMV + HHV7**	1(1.1)	–	1 (2)	1.000
**Negative for all**	35(36.8)	16 (37.2)	19 (36.5)	1.000
**Positive for 1 or more**	60(63.2)	27 (62.8)	33 (63.5)	1.000

The patients were divided into two main groups according to the type of leukemia: (1) patients with AML (*N* = 43) and (2) patients with ALL (*N* = 52). A *p* value of < 0.05 was considered to be statistically significant

Among all the patients, CMV was the most frequently identified HHVs 48.4% (46/95), followed by HHV-6 26.3% (25/95), HHV-7 14.7% (14/95) and EBV 8.4% (8/95). Fifteen patients (15.8%) were infected with CMV and HHV-6, of whom five tested positive for HHV6 first, three for CMV first, and seven for both simultaneously. Five patients (5.3%) were infected with CMV and HHV-7, of whom two tested positive for CMV first and one for HHV-7 first and two for both simultaneously. One patient (1%) was co-infected with both EBV and HHV-7 at the same time. Two patients (2.1%) were infected with CMV, HHV-6 and HHV-7, of whom one tested positive for both HHV-6 and HHV-7 first and one patient with all three viruses simultaneously. Two patients (2.1%) were infected with CMV, EBV and HHV-6, of whom one tested positive simultaneously for CMV and EBV first and one patient with both EBV and HHV-6. One patient (1%) was infected with three viruses, EBV and HHV-7 simultaneously then HHV-6. Finally, one patient (1%) was co-infected with EBV and HHV-7 then CMV. Out of the overall 84 HHVs distinct infections (from 60 patients), 61 were single infections and 23 were co-infections**.** For a single patient, the total number of any HHVs positive infections ranged from 1 to 4 (45 patients had only one positive detection, 8 patients had 2 positive distinct detections, 5 patients had 3 distinct positive detections and 2 patients had 4 positive distinct detections). Follow-up samples were collected; for an individual patient, the total number of HHVs positive plasma samples detected ranged from 1 to 9 (17 patients had 1 positive sample, 16 patients had 2, 6 patients had 3, 5 patients had 4, 16 patients had ≥5 positive samples). All the sequences of the first PCR products confirmed their viral identity when compared to NCBI web site sequences. The overall prevalence of CMV DNAemia (≥57.1 copies/mL) and high-level CMV DNAemia (≥1000 copies/mL) were obtained in 65.5% (38/58) and 34.5% (20/58) of viral detections.

Samples with high-level CMV DNAemia were detected both in post-remission and at salvage phase of chemotherapy and not before chemotherapy.Twelve recurrent infections of CMV DNAemia were detected. A second recurrent infection was reported in eight patients; half of them after remission chemotherapy (consolidation) and a half after salvage chemotherapy and a third recurrent infection was reported at salvage chemotherapy in two patients who had previously tested positive; one before chemotherapy and after consolidation and one after induction and consolidation chemotherapy. Three recurrent infections of HHV-6 DNAemia were present in two patients; one had the first episode at post-remission, then a second one at salvage chemotherapy and the other patient had two recurrent infections; one at diagnosis then one at induction and another one at post-remission. Only one recurrent infection of HHV-7 DNAemia was present in one AML patient.

Table 3 demonstrates the prevalence of HHVs classified by phases of chemotherapy. According to each period, CMV DNAemia was detected in 8.4, 14.7, 27.1, and 51.7% before chemotherapy, at post-induction, post-remission, and after salvage, respectively. The prevalence of CMV DNAemia was significantly higher at salvage than before chemotherapy or post-induction (p < 0.05). HHV-6 DNAemia was detected in 1, 3.15, 2.8 and 7% before chemotherapy, at post-induction, post-remission, and after salvage, respectively.

**Table 3:** Presence of herpesviruses DNA according to the different chemotherapy phases as detected by multiplex PCR

Viral detection	Before chemotherapy ***N*** = 95 (%)	Post- induction ***N*** = 95 (%)	Post -remission ***N*** = 70 (%)	After salvage ***N*** = 29 (%)	All periods ***N*** = 289 (%)
**CMV**	8 (8.4)	14 (14.7)	19 (27.1)	15 (51.7)	56 (19.4)
**EBV**	1 (1)	3 (3.15)	2 (2.8)	2 (7)	8 (2.7)
**HHV-6**	1 (1)	8 (8.4)	9 (12.9)	10 (34.5)	28 (9.7)
**HHV-7**	1 (1)	6 (6.3)	3 (4.2)	5 (17.2)	15 (5.2)
**Patients negative for all**	86 (90.5)	74 (78)	46 (65.7)	7 (24.1)	213 (73.7)
**Patients positive for 1 or more detection**	9 (9.5)	21 (22)	24 (34.3)	22 (75.9)	76 (26.3)

The prevalence of viral infections during the different phases of chemotherapy: before chemotherapy, post-induction, post-remission and after salvage

The prevalence of HHV-6 DNAemia was significantly higher at different stages of chemotherapy than before chemotherapy (*p* < 0.05) and higher at salvage than at post-induction or at post-remission (*p* < 0.05). EBV DNAemia was detected rarely in 1, 3.15, 2.8 and 7% before chemotherapy, at post-induction, post-remission, and after salvage, respectively. HHV-7 DNAemia was detected in 1, 6.3, 4.2 and 17.2% before chemotherapy, at post-induction, post-remission, and after salvage, respectively. The prevalence of HHV-7 DNAemia was significantly higher at salvage than before chemotherapy or at post-induction (*p* < 0.05).

### Risk factors for herpes infections

In the univariate analysis, risk factors for CMV infection were the presence of relapse (*p* = 0.033, OR = 2.455), receiving salvage chemotherapy (*p* = 0.027, OR = 4.885) and the occurrence of HHV-6 infection (*p* = 001, OR = 5.043). Presence of relapse (*p* = 0.014, OR = 3.2), receiving salvage chemotherapy (*p* = 0.007, OR = 3.656) and the occurrence of CMV infection were significantly associated with increased risk of HHV-6 infection (*p* = 001, OR = 5.043).

Interestingly, 76% of HHV-6 infections were followed by a CMV infection. HHV-7 infection was associated with an increased risk of EBV infection with borderline significance and vice versa (*p* = 0.09). Other potential risk factors, such as gender, age, type of leukemia and response to induction were not found to be risk factors for CMV, EBV, HHV-6 and HHV-7 infections. After binary logistic analysis, only HHV-6 infection (OR 4.205, *p* = 0.008) was identified as an independent risk factor for CMV infection while, salvage treatment (OR 2.887, *p* = 0.04) and CMV infection (OR 4.265, *p* = 0.007) remained as independent risk factors for HHV-6 infection (Table 4**).**

**Table 4:** Risk factors for the development of viral infection. Patient-Level: (at least one positive PCR-result)

Risk Factors (Univariate analysis)		CMV infection			EBV infection			HHV-6 infection			HHV7- infection		
Patients Data		Negative ***N*** = 49 (%)	Positive ***N*** = 46 (%)	***P***	Negative ***N*** = 87 (%)	Positive ***N*** = 8 (%)	***P***	Negative ***N*** = 70 (%)	Positive ***N*** = 25 (%)	***P***	Negative ***N*** = 81(%)	Positive ***N*** = 14 (%)	***P***
**Gender**	Male	31 (49.2)	32 (50.8)	0.516	56 (88.9)	7 (11.1)	0.260	44 (69.9)	19 (30.1)	0.233	54 (58.7)	9 (14.3)	0.862
	Female	18 (56.2)	14 (43.8)		31 (96.9)	1 (3.1)		26 (81.2)	6 (18.8)		27 (84.4)	5 (15.6)	
**Age groups (years)**	< 16	26 (53.1)	23 (46.9)	0.765	44 (89.8)	5 (10.2)	0.518	35 (71.4)	14 (28.6)	0.606	40 (81.6)	9 (18.4)	0.303
	≥ 16	23 (50)	23 (50)		43 (93.5)	3 (6.5)		35 (76.1)	11 (23.9)		41 (89.1)	5 (10.9)	
**Type of leukemia**	AML	18 (41.9)	25 (58.1)	0.085	41 (95.3)	2 (4.7)	0.286	32 (74.4)	11 (25.6)	0.882	37 (86)	6 (14)	0.845
	ALL	31 (59.6)	21 (40.4)		46 (88.5)	6 (11.5)		38 (73.1)	14 (26.9)		44 (84.6)	8 (15.4)	
**Response to induction**	Non-complete remission	11 (44)	14 (56)	0.377	23 (92)	2 (8)	0.930	20 (80)	5 (20)	0.403	22 (88)	3 (12)	0.653
	Complete remission	38 (54.3)	32 (45.7)		64 (91.4)	6 (8.6)		50 (71.4)	20 (28.6)		59 (84.3)	11 (15.7)	
**Disease status (**at the last follow-up)	Remission	33 (61.1)	21 (38.9)	**0.033**	51 (94.5)	3 (5.5)	0.248	45 (83.3)	9 (16.7)	**0.014**	48 (88.8)	6 (11.2)	0.253
	Relapse/Refractory	16 (39)	25 (61)		36 (87.8)	5 (12.2)		25 (61)	16 (39)		33 (80.5)	8 (19.5)	
**Salvage**	No received	39 (59.1)	27 (31.9)	**0.027**	62 (93.9)	4 (6.1)	0.211	54 (81.8)	12 (18.2)	**0.007**	59 (89.4)	7 (10.6)	0.087
	Received	10 (34.5)	19 (65.5)		25 (86.2)	4 (13.8)		16 (55.2)	13 (44.8)		22 (75.9)	7 (24.1)	
**Outcome**	Death	7 (41.2)	10 (58.8)	0.344	15 (88.2)	2 (11.8)	0.631	12 (70.6)	5 (29.4)	0.749	15 (88.2)	2 (11.8)	0.703
	Alive	42 (53.8)	36 (46.2)		72 (92.3)	6 (7.7)		58 (74.4)	20 (25.6)		66 (84.6)	12 (15.4)	
**Virus**	CMV infection	–	–	–	43 (93.5)	3 (6.5)	0.716	27 (58.7)	19 (41.3)	**0.001**	38 (82.6)	8 (17.4)	0.479
	EBV infection	5 (62.5)	3 (37.5)	0.518	–	–	**–**	5 (62.5)	3 (37.5)	0.430	5 (62.5)	3 (37.5)	0.092
	HHV-6 infection	6 (24)	19 (76)	**0.001**	22 (88)	3 (12)	0.430	–	–	–	22 (88)	3 (12)	0.754
	HHV-7 infection	6 (42.8)	8 (57.2)	0.479	11 (78.6)	3 (21.4)	0.092	11 (78.6)	3 (21.4)	0.754	–	–	–
**Multivariate analysis for viral infection**		**Factors**		**Coefficient (B)**		**Odds Ratio (OR)**			**95% Confidence Interval**			**P**	
**CMV infection**		HHV-6 infection		1.436		4.205			1.450–12.198			**0.008**	
**HHV-6 infection**		Salvage received		1.060		2.887			1.048–7.955			**0.040**	
		CMV infection		1.450		4.265			1.474–12.345			**0.007**	

Univariate logistic regression analysis of risk factors for HHVs (EBV, CMV, HHV-6 and HHV-7) infections were performed, showing the association between viral infections and patient’s demographic and clinical characteristics using the Chi-square (X^2^) test or Fisher’s exact test. Statistically significant association was considered if *p*-value *<* 0.05 after multivariate analysis (significant *p* are bolded)

### Association between viral co-infection and clinical presentation

When comparing infections involving only single herpes viral infection with infections involving herpes viral co-infection, no statistically significant differences were observed with respect to age groups (< 16 and ≥ 16 years), type of leukemia**,** disease status and therapy phase. However, male gender was significantly higher in viral co-infection compared with single infection (*p* = 0.007). Detections involving herpes viral co-infections were associated with higher rates of mortality compared with a single viral infections, with borderline significance (*p* = 0.058), but this finding was not time-related**.** Table 5 shows that the presence of severe lymphopenia (ALC < 500/μL) was significantly higher in co-infections compared with mono-viral infections (*p* < 0.001). Herpes viral co-infection group compared with only single herpes viral infection group had more often rash, pneumonia, bacteremia and fungal co-infection (*p* = 0.04, *p* = 0.008, *p* < 0.001, *p* = 0.038, respectively)**.**

**Table 5:** Relation between demographic and clinical characteristics of patients with mono-viral infection and viral co-infection

Variables	Active viral infection (***N*** = 84) No. (%)	Active mono-viral infection (***N*** = 61) No. (%)	Active viral co-infection (***N*** = 23) No. (%)	***P***
**Sex**				
Male	54 (64.3)	34 (55.7)	20 (86.9)	**0.007**
Female	30 (35.7)	27 (44.3)	3 (13)	
**Age (years)**				
< 16	49(58.3)	37 (60.7)	12 (52.2)	0.482
≥ 16	35 (41.7)	24 (39.3)	11 (47.8)	
**Type of leukemia**				
AML	36 (42.9)	25 (41)	11 (47.8)	0.572
ALL	48 (57.1)	36 (59)	12 (52.2)	
**Status of disease**				
Newly diagnosed	9 (10.7)	7 (11.5)	2 (8.7)	0.443
Remission	54 (64.3)	41 (67.2)	13 (56.5)	
Refractory or relapse	21 (25)	13 (21.3)	8 (34.8)	
**Chemotherapy received**				
Before chemotherapy	9 (10.7)	7 (11.5)	2 (8.7)	0.607
Post-induction	21 (25)	13 (21.3)	8 (34.8)	
Post-remission	28 (33.3)	22 (36)	6 (26.1)	
Post-salvage	26 (31)	19 (31.1)	7 (30.4)	
**Clinical outcome**				
Alive	72 (85.7)	55 (90.2)	17 (74)	0.058
Dead	12 (14.3)	6 (9.8)	6 (26)	
**Fever**	56 (66.6)	41 (67.2)	15 (65.2)	0.863
**Severe neutropenia**	48 (57.2)	32 (52.5)	16 (69.5)	0.158
**Prolonged neutropenia**	38 (45.3)	27 (44.3)	11 (47.8)	0.209
**Severe lymphopenia**	45 (53.5)	25 (41)	20 (87)	**< 0.001**
**Anemia**	28 (33.3)	20 (32.8)	8 (43.8)	1.000
**Thrombocytopenia**	29 (34.5)	21 (34.4)	8 (43.8))	0.986
**Skin rash**	17 (20.2)	8 (13.1)	9 (39.1)	**0.04**
**Mucositis**	5 (6)	3 (5)	2 (8.7)	0.514
**Pneumonia**	17 (20)	8 (13.1)	9 (39)	**0.008**
**Gastroenteritis**	10 (11.9)	7 (11.5)	3 (13)	0.843
**Hepatitis**	12 (14.3)	7 (11.5)	5 (21.7)	0.231
**Conjunctivitis**	2 (2.4)	0 (0)	2 (8.7)	–
**CNS disorders**	7 (8.3)	3 (5)	4 (17.4)	0.065
**Other infection**				
**BSI**	16 (19)	6 (9.8)	10 (43.5)	**< 0.001**
**IFI**	14 (16.6)	7 (11.5)	7 (30.4)	**0.038**

Viral infections were divided into two groups based on the number of viruses detected: active mono-viral infections and co-infections

Severe neutropenia [ANC < 500/μL]; Prolonged neutropenia [if it persisted over 7 days]; Severe lymphopenia [ALC < 500/μL]; Anemia [hemoglobin < 11 g/L], Thrombocytopenia [platelet count < 100 10 ^3^/ μL]; *CNS disorders* Central Nervous System disorders, *BSI* Blood Stream Infection, *IFI* Invasive Fungal Infection

Statistically significant *p* are bolded

The detailed relationship between CMV mono-viral infection and CMV co-infection and clinicopathological parameters are presented in Table 6**.**

**Table 6:** Relation between demographic and clinical characteristics of patients with CMV mono-viral infection and CMV viral co-infection

Variables	No active CMV infection (***N*** = 26) No. (%)	Active CMV mono-infection (***N*** = 39) No. (%)	Active CMV co-infection (***N*** = 19) No. (%)	***P***
Sex				
**Male**	15 (57.7)	23 (59)	16 (84.2)	0.119
**Female**	11 (42.3)	16 (41)	3 (15.8)	
**Age (years)**				
< 16	17 (65.4)	22 (56.4)	10 (52.6)	0.655
≥ 16	9 (34.6)	17 (43.6)	9 (47.4)	
**Type of Leukemia**				
AML	6 (23)	21 (53.8)	9 (47.4)	**0.044**
ALL	20 (76.9)	18 (46.2)	10 (52.6)	
**Status of disease**				
Newly diagnosed	1 (4)	6 (15.4)	2 (10.5)	0.342
Remission	20 (76.9)	24 (61.5)	10 (52.6)	
Refractory or relapse	5 (19.1)	9 (23)	7 (36.8)	
**Chemotherapy received**				
Before chemotherapy	1 (3.8)	6 (15.4)	2 (10.5)	0.802
Post-induction	7 (26.9)	10 (25.7)	4 (21)	
Post-remission	8 (30.8)	13 (33.3)	7 (36.8)	
Post-salvage chemotherapy	10 (38.5)	10 (25.6)	6 (31.6)	
**Clinical outcome**				
Alive	24 (92.3)	34 (87.2)	14 (73.7)	0.198
Dead	2 (7.7)	5 (12.8)	5 (26.3)	
**Fever**	16 (61.5)	28 (71.8)	12 (63.1)	0.646
**Severe neutropenia**	11 (42.3)	24 (61.5)	13 (68.4)	0.163
**Prolonged neutropenia**	8 (30.8)	20 (51.3)	10 (52.6)	0.752
**Severe lymphopenia**	11 (42.3)	18 (46.2)	16 (84.2)	**0.009**
**Anemia**	7 (26.9)	15 (38.5)	6 (31.6)	0.507
**Thrombocytopenia**	7 (26.9)	16 (41)	6 (31.6)	0.364
**Skin rash**	2 (7.7)	6 (15.4)	8 (42.1)	**0.011**
**Mucositis**	1 (3.8)	2 (5.2)	2 (10.5)	–
**Pneumonia**	2 (7.7)	7 (17.9)	8 (42.1)	**0.016**
**Gastroenteritis**	–	7 (17.9)	3 (15.8)	0.084
**Hepatitis**	2 (7.7)	5 (12.8)	5 (26.3)	0.198
**Conjunctivitis**	–	2 (5.2)	–	–
**CNS disorders**	1 (3.8)	2 (5.2)	4 (21)	0.073
**Other infection**				
**BSI**	4 (15.4)	4 (10.3)	8 (42.1)	**0.013**
**IFI**	2 (7.7)	5 (12.8)	7 (36.8)	**0.024**

Viral infections were divided into three groups (1) no active CMV infection, (2) active mono-viral infection with only CMV and no other HHVs infection and (3) active co-infection with CMV and other HHVs

Severe neutropenia [ANC < 500/μL]; Prolonged neutropenia [if it persisted over 7 days]; Severe lymphopenia [ALC < 500/μL]; Anemia: [hemoglobin < 11 g/L], Thrombocytopenia [platelet count < 100 10 ^3^ /μL]; *CNS disorders* Central Nervous System disorders, *BSI* Blood Stream Infection, *IFI* Invasive Fungal Infection

Statistically significant *p* are bolded

No active CMV infection, Active CMV mono-viral infection and active CMV co-infection groups had common characteristics including age groups**,** status of disease, and phase of therapy. However, no active CMV infection group was more often treated for acute lymphoblastic leukemia (ALL) compared to single CMV infection (*p* = 0.044)**.** The presence of active CMV co-infection was more associated with high-risk clinical parameters including severe lymphopenia (*p* = 0.009), rash (*p* = 0.011), pneumonia (*p* = 0.016), bacteremia (*p* = 0.013) and fungal infection (*p* = 0.024) than with patients with active CMV mono-viral infection.

## Discussion

There have been limited studies on herpes viral infection in non-transplant acute leukemia patients undergoing chemotherapy and the majority of them have focused on CMV, with very little regard for other HHVs. The current study aimed, therefore, to investigate the frequency of six HHVs at different phases of chemotherapy and to identify their possible interactions during co-infections. We also studied the potential risk factors for viral infections and their impact on the clinical outcome during coexistence.

In contrast to developed countries, where HHVs seroprevalence and occurrence are well documented, there is only scarce information available in Tunisia [15, 16].To our knowledge, the seroprevalence of HHVs in patients with acute leukemia has not been reported previously in Tunisia. According to our findings, the pre-chemotherapy serologic status for HHVs (EBV, VZV, CMV, HSV, HHV-6) were very high, ranging from 81 to 97.9%. Thus, most of the patients were seropositive before chemotherapy, and most probably viral DNAemia during chemotherapy was due to reactivation or reinfection under immunosuppressive conditions rather than primo-infection.

The DNA detection of CMV, HHV-6, HHV-7 and EBV were 19.4, 9.7, 5.2 and 2.7% respectively. The data concerning HHVs infections observed in literature are very heterogeneous in the non-transplant setting. The rate of CMV infection or recurrence varied from 2 to 67% among reported patients with hematological malignancies receiving non-transplant treatment [2, 16–21]. Similar to our study, almost the same percentage of HHV-6 infection was reported in previous studies in acute leukemia patients [17, 18]. However, our frequencies were lower than those in a previous study which reported a high incidence of CMV, HHV-6 and EBV in patients receiving cytotoxic chemotherapy for adult T cell leukemia [19]. Rizk and collaborators have recently reported a higher incidence of EBV, HHV-6 and HHV-7 in newly diagnosed children with acute lymphoblastic leukemia [20]. In contrast, another study carried out by Persson and al did not detect any HHV-6 or HHV-7 DNA in any of the febrile episodes in neutropenic patients treated with cytotoxic chemotherapy [21]. In the current study, HSV and VZV DNA was not detected in plasma samples indicating that DNAemia are not common in this group of patients suggesting that DNAemia might be rarely observed in recurrent infections in immunocompromised hosts [22, 23]. Patients who are present from geographic regions with a high seroprevalence of either virus in both developing and developed countries are at a higher risk of viral reactivation and symptomatic and asymptomatic screening should be tailored to the present increased risk [8].

Discrepancies among these studies could be attributed to several reasons including differences in methodology, clinical and epidemiological characteristics, frequency of virological monitoring, type of specimen and the wide spectrum of technique sensitivity.

Our data showed that HHVs detections were higher after induction, after post-remission, and at salvage chemotherapy than before chemotherapy confirming the recurrence of these viruses after chemotherapy. This seems to be related to specific defects of immune function detectable at diagnosis of leukemia and arising during treatment [17, 18, 24]. Despite more intensive chemotherapy in the prior phases, the risk of infection does not totally decrease once achieving remission as patients will have persistent immunosuppression. Reconstitution of B lymphocytes and Natural killer cells occurs early while T cell reconstitution shows delayed recovery of both T helper and T suppressor cells [25]. Higher risk of herpes viral infection during post-remission phases of chemotherapy in acute leukemia patients was also previously reported in adult and pediatric populations especially infected with CMV and HHV-6 infections [17, 19, 26–28].

Our results showed that HHVs infections did not seem to confer protection against chemoresistance and hematologic disease relapse. It was found in our study that the high incidence of CMV and HHV-6 viral infection occurred during post-remission but also this risk increases further with relapse of disease, consistent with other previous studies [17, 24]. Furthermore, the use of salvage chemotherapy (relapsed/refractory) was associated with the significant increase in CMV and HHV-6 infections in univariate analysis but not in multivariate analysis in CMV infections. This increase might be due to exposure to more selective suppressive chemotherapy such as fludarabine and the use of high-dose of steroids which depresses CD4 T-lymphocyte leading to a prolonged lymphopenia or T-cell dysfunction [6, 7, 28–31]. However, paradoxical recent data suggest that CMV replication may protect against AML relapse after allo-HSCT [32]. Whether CMV reactivation can protect against relapse after allo-HSCT for other hematological malignancies patients is controversial [33, 34]. More research is further needed to better understand these enigmatic observations.

This study allowed us to recognize multiple infections in 28.4% (27/95) of acute leukemia patients. The majority of the multiple infected patients had co-infections (23/27). Sixteen patients had combined CMV-HHV6 infections. Both active infections were significantly associated (p < 0.001) which is consistent with previous studies examining the relationships and the effects of HHV combinations, particularly in the context of solid organ transplant SOT recipients. β-HHVs were found to transactivated each other; while CMV infection appeared to trigger HHV-6 and/or HHV-7 co-infection and vice versa [6, 35–39]. Recent studies have also shown that the activation of HHV-6 can predispose allo-HSCT recipients for CMV infection, and suggest that HHV-6 infection precedes CMV activation in time [7, 38–41]. Similarly, in the current study, prior HHV-6 infection was associated with higher rates of CMV activation.

Based on these results, the time of CMV activation could be predicted based on the timing of HHV-6 infection. HHV-6 is known to have immunomodulatory and immunosuppressive effects that could predispose patients to CMV reactivation/infection [7, 30]. Although, in a previous study in acute leukemia patients, the association between HHV-6 and CMV was only found in saliva samples but not in blood samples [24]. The difference in the results might be due to the limited number of events included in that latter study which does not allow them to draw definitive conclusions on this matter. Data from several studies suggested a potential indirect effect of HHV-7 to increase rates of CMV and HHV-6 reactivations and disease in HSCT [12, 40, 41] and SOT recipients [37, 42], but this is not a consistent finding in all studies [11, 43]. Similarly to our study, no relationship was found between CMV and HHV-7 or between HHV-6 and HHV-7. The relationships between β and γ-HHVs infections remain poorly understood, but potential evidence of viral cooperation has been reported in HSCT recipients; prior infection with EBV was shown to promote HHV-6 [26] and CMV infections [44–46]. In the present study, no relationship was found between CMV and EBV or between EBV and HHV-6, although a favourable trend was observed between HHV-7 and EBV (p = 0.09). The limited number of included events minimizes the significance of our analysis. But, Wang and coworkers have reported a high incidence of secondary infections in children with simultaneously CMV and EBV primary infection [47]. In SOT recipients, Sanchez-Ponce and colleagues had reported that EBV and HHV-7 co-infections were present in the majority of co-infections episodes (62%) and suggested that this co-infection was associated with graft rejection [48]. The significant association between EBV and HHV-7 was also described in Hodgkin’s disease [49] but to our knowledge not in leukemic patients undergoing chemotherapy. However, the association between HHVs may just be the result of state of a severe immunosuppression that triggers viral co-activation, and not necessarily because of their interaction. Evidence related to the severity of co-infection compared to a single viral infection is conflicting and unclear in several previous reports. Evidence for a correlation between the reactivation of HHVs and different clinical manifestations has been suggested mainly from studies on SOT recipients [42, 46], allo-HSCT [50, 51] or after autologous peripheral blood stem cell transplantation [52]. Nevertheless, very little is known about the biologic and clinical consequences of the interaction between different HHVs in non-transplant acute leukemia patients undergoing chemotherapy. Based on our statistical analysis the prevalence of HHV co-infections (not the load) was correlated with more significant clinical symptoms than mono viral infections suggesting that the association of these co-infections at any level with clinically relevant parameters may be sufficient to modify the clinical presentation and pathogenesis of these viruses. However, the clinical relevance of HHVs viral infections need to be evaluated by quantitative real-time PCR. These techniques would be much more useful for interpreting the contradictions produced by attributing greater or lesser severity to co-infection.

In this work, the highest frequencies of viral co-infections were found in men although the specific reasons underlying this effect remains unknown. Several studies have shown that behavioral factors and physiological differences between males and females cause dimorphic responses to infection. The intensity and prevalence of viral infections were found to be higher in males, whereas females typically display reduced susceptibility to viral infections because they often mount stronger immune responses than males [53].

According to our study, herpes viral co-infection showed a more significant association with profound lymphopenia (ALC < 500/μL), pneumonia, opportunistic infections (bacteremia and invasive fungal infection) than with single infection and a borderline association with increased risk of death. Diseases that cause lymphopenia are typically associated with an increased predisposition to various infections, either directly as a result of the lymphopenia-associated immune suppression or because of the underlying disease. There seems to be a strong relationship between the degree of lymphopenia and the acquisition of multiple opportunistic infections. Similar findings were reported in SOT settings, where pre- or post-transplant ALC, as well as specific lymphocyte subsets, were associated with the development of opportunistic infections following transplantation [54–57]. In patients with hematological malignancies or prior HSCT, lymphocytopenia was significantly associated with serious CMV pneumonia [5] which leads more frequently to death than other infections. In fact, Caifeng Yue et al. showed that CMV and respiratory syncytial virus (RSV) co-infection facilitates the development of severe pneumonia [58]. Direct herpesvirus mediated tissue injury (e.g. pneumonia) may be under-recognized or misattributed to other causes in non-transplant setting, which in turn would predispose patients to greater toxicity from other insults. Viremia with HHVs may have indirect effects due to increased production of pro-inflammatory and immunomodulatory cytokines leading to the pathogenesis of important complications after HSCT [59, 60]. Since HHV-6 and CMV each have similar pathogenic effects, it follows that active co-infection of both viruses might be associated with worse clinical outcomes than in mono-viral infection; viremia of CMV and HHV-6 has been associated with increased risk of bacterial and fungal infections as demonstrated similarly in this study and in previous studies [59–62] . The results from the present study could indicate that individuals with CMV and other herpes viral co-infections should benefit from additional medical attention because of their increased frequency of severe lymphopenia, pneumonia, rash and secondary infections, which are a significant cause of morbidity and mortality in leukemic patients undergoing chemotherapy treatment [63, 64]. Care must be taken in case our findings are extrapolated to other settings. This study had limitations such as the small number of patients enrolled, the monocentric type of study, and the irregularity of sampling intervals. Establishing the significance of HHVs is a complex process, especially in the immunocompromised patient. HHVs may represent asymptomatic or latent infection. HHVs are probably on the margin of the detection limit by current methods, making it hard for different labs to achieve the same conclusion. Larger epidemiological studies and quantitative detection techniques are needed to confirm the role of viral co-infections in acute leukemia and how they correlate with clinical severity. Quantitative PCR would help to determine the declining or rising of viral load that could be important for pre-emptive antiviral therapy. In the present study, there was no biopsy available for the CMV DNA detection to confirm the diagnosis of end organ-disease. Therefore, the clinical hypothesis of end organ-disease was supported only by the presence of CMV viral DNA in plasma associated with symptomatic clinical manifestation. Testing for viral detection in other compartments (e.g., Bronchoalveolar lavage fluid, cerebrospinal fluid) may be important in future studies.

## Conclusion

This study showed that HHVs infections were high in acute leukemia patients who were receiving chemotherapy. We demonstrated the presence and the possible clinical relevance of HHVs coinfections. CMV/HHV6 co-infections were the most frequent co-infections. The clinical outcome of HHVs co-infection was more severe in HHVs single infection which suggests that viruses were frequently involved in the complications after chemotherapy. Future prospective studies in larger acute leukemia cohorts should be performed to better clarify the spectrum of these viral infections, to define the mechanism underlying the potential evidence of viral cooperation and to determine if prevention or treatment of reactivating viruses leads to improve outcomes.

## Supplementary information

**Additional file 1: Figure S1.** Specificity of herpesviruses multiplex PCR. Agarose gel electrophoresis of herpesviruses multiplex PCR showed that a unique PCR product of the expected size was amplified in each positive control. MM: 100pb (base pairs) molecular weight marker and Negative Control (No band).

## Data Availability

Data are available from the corresponding author on reasonable request.

## Abbreviations

ALC: Absolute lymphocyte count
ALL: Acute lymphoblastic leukemia
allo-HSCT: Allogeneic hematopoietic stem cell transplant
AML: Acute myeloid leukemia
ANC: Absolute neutrophil count
CMV: Cytomegalovirus
CR: Complete remission
EBV: Epstein-Barr virus
HHV-6: Human herpesvirus 6
HHV-7: Human herpesvirus 7
HHV-8: Human herpesvirus 8
HHVs: Human herpesviruses
HSV: Herpes simplex virus
IFI: Indirect immunofluorescent techniques
IgG: Immunoglobulin G
IgM: Immunoglobulin M
OR: Odds ratio
PCR: Polymerase Chain Reaction
SOT: solid organ transplant
VZV: Varicella -zoster virus
α-HHVs: Alpha-human herpesviruses
β-HHVs: Beta-human herpesviruses
γ-HHVs: Gamma- human herpesviruses

## References

[1] Öhrmalm L, Wong M, Aust C, Ljungman P, Norbeck O, Broliden K, Tolfvenstam T. Viral Findings in Adult Hematological Patients with Neutropenia. PLoS One. 2012;7:e36543. 10.1371/journal.pone.0036543 PMC3343003 22570724

[2] Marchesi F, Pimpinelli F, Ensoli F, Mengarelli A (2018). Cytomegalovirus infection in hematologic malignancy settings other than the allogeneic transplant. Hematol Oncol.

[3] Sehrawat S, Kumar D, Rouse BT (2018). Herpesviruses: Harmonious Pathogens but Relevant Cofactors in Other Diseases?. Front Cell Infect Microbiol.

[4] Rahbarimanesh A, Ehsani M, Karahroudi M, Rashidi A, Aghajani M, Meysami A, Shahgholi E, Mehrvar A, Tashvighi M, Keyvani H (2015). Cytomegalovirus Disease in Children With Acute Lymphoblastic Leukemia in the Nontransplant Setting: Case Series and Review of the Literature. J Pediatr Hematol Oncol.

[5] Torres HA, Aguilera E, Safdar A, Rohatgi N, Raad SC, Luna M, Kontoyiannis DP, Chemaly RF (2008). Fatal cytomegalovirus pneumonia in patients with haematological malignancies: an autopsy-based case-control study. Clin Microbiol Infect.

[6] Amanati A, Shakibazad N, Pourabbas B, Nowroozzadeh MH, Zareifar S, Zekavat OR (2018). Acute Progressive Visual Loss in a Case of Acute Myeloid Leukemia: Challenges in the Utility of Molecular Tests in Early Diagnose of Cytomegalovirus Retinitis. Case Rep Med.

[7] Harada K, Sekiya N, Ikegawa S, Sasaki S, Kobayashi T, Ohashi K (2019). Cytomegalovirus meningitis in a patient with relapsed acute myeloid leukemia. Int J Hematol.

[8] Dixon SB, Lane A, O'Brien MM, Burns KC, Mangino JL, Breese EH, Absalon MJ, Perentesis JP, Phillips CL. Viral surveillance using PCR during treatment of AML and ALL. Pediatr Blood Cancer. 2018;65. 10.1002/pbc.26752. 10.1002/pbc.26752 28792686

[9] Wade JC. Viral infections in patients with hematological malignancies. Hematology Am Soc Hematol Educ Program. 2006;2006:368–74. 10.1182/asheducation-2006.1.368 17124085

[10] Wingard JR, Hsu J, Hiemenz JW (2010). Hematopoietic stem cell transplantation: an overview of infection risks and epidemiology. Infect Dis Clin N Am.

[11] Humar A, Asberg A, Kumar D, Hartmann A, Moussa G, Jardine A, Rollag H, Mouas H, Gahlemann CG, Pescovitz MD, Group Vs (2009). An assessment of herpesvirus co-infections in patients with CMV disease: correlation with clinical and virologic outcomes. Am J Transplant.

[12] Zawilinska B, Kopec J, Szostek S, Piatkowska-Jakubas B, Skotnicki AB, Kosz-Vnenchak M (2011). Lymphotropic herpesvirus DNA detection in patients with active CMV infection - a possible role in the course of CMV infection after hematopoietic stem cell transplantation. Med Sci Monit.

[13] Tanaka T, Kogawa K, Sasa H, Nonoyama S, Furuya K, Sato K (2009). Rapid and simultaneous detection of 6 types of human herpes virus (herpes simplex virus, varicella-zoster virus, Epstein-Barr virus, cytomegalovirus, human herpes virus 6A/B, and human herpes virus 7) by multiplex PCR assay. Biomed Res.

[14] Ljungman P, Boeckh M, Hirsch HH, Josephson F, Lundgren J, Nichols G, Pikis A, Razonable RR, Miller V, Griffiths PD (2017). Disease Definitions Working Group of the Cytomegalovirus Drug Development F: Definitions of Cytomegalovirus Infection and Disease in Transplant Patients for Use in Clinical Trials. Clin Infect Dis.

[15] Hannachi N, Marzouk M, Harrabi I, Ferjani A, Ksouri Z, Ghannem H, Khairi H, Hidar S, Boukadida J (2011). Seroprevalence of rubella virus, varicella zoster virus, cytomegalovirus and parvovirus B19 among pregnant women in the Sousse region, Tunisia. Bull Soc Pathol Exot.

[16] Hannachi N, Boughammoura L, Marzouk M, Tfifha M, Khlif A, Soussi S, Skouri H, Boukadida J (2011). Viral infection risk in polytransfused adults: seroprevalence of seven viruses in central Tunisia. Bull Soc Pathol Exot.

[17] Nefzi F, Ben Salem NA, Khelif A, Feki S, Aouni M, Gautheret-Dejean A (2015). Quantitative analysis of human herpesvirus-6 and human cytomegalovirus in blood and saliva from patients with acute leukemia. J Med Virol.

[18] Seror E, Coquerel B, Gautheret-Dejean A, Ballerini P, Landman-Parker J, Leverger G, Schneider P, Vannier JP (2008). Quantitation of Human herpes virus 6 genome in children with acute lymphoblastic leukemia. J Med Virol.

[19] Ogata M, Satou T, Kawano R, Yoshikawa T, Ikewaki J, Kohno K, Ando T, Miyazaki Y, Ohtsuka E, Saburi Y (2011). High incidence of cytomegalovirus, human herpesvirus-6, and Epstein-Barr virus reactivation in patients receiving cytotoxic chemotherapy for adult T cell leukemia. J Med Virol.

[20] Anies Rizk M, Darwish A (2019). Study of Epstein Barr virus, Human Herpes 6 and Human Herpes 7 in Children with Acute Lymphoblastic Leukemia. Int J Curr Microbiol App Sci.

[21] Persson L, Dahl H, Linde A, Engervall P, Vikerfors T, Tidefelt U (2003). Human cytomegalovirus, human herpesvirus-6 and human herpesvirus-7 in neutropenic patients with fever of unknown origin. Clin Microbiol Infect.

[22] Patrick K, Ali M, Richardson SE, Gassas A, Egeler M, Krueger J, Lowry J, Allen U, Schechter T (2015). The yield of monitoring for HSV and VZV viremia in pediatric hematopoietic stem cell transplant patients. Pediatr Transplant.

[23] Zuckerman RA (2009). The clinical spectrum of herpes simplex viremia. Clin Infect Dis.

[24] Faten N, Agnes GD, Nadia BF, Nabil AB, Monia Z, Abderrahim K, Henri A, Salma F, Mahjoub A (2012). Quantitative analysis of human herpesvirus-6 genome in blood and bone marrow samples from Tunisian patients with acute leukemia: a follow-up study. Infect Agent Cancer.

[25] El-Chennawi FA, Al-Tonbary YA, Mossad YM, Ahmed MA (2008). Immune reconstitution during maintenance therapy in children with acute lymphoblastic leukemia, relation to co-existing infection. Hematology.

[26] Jain R, Trehan A, Mishra B, Singh R, Saud B, Bansal D (2016). Cytomegalovirus disease in children with acute lymphoblastic leukemia. Pediatr Hematol Oncol.

[27] Capria S, Gentile G, Capobianchi A, Cardarelli L, Gianfelici V, Trisolini SM, Foa R, Martino P, Meloni G (2010). Prospective cytomegalovirus monitoring during first-line chemotherapy in patients with acute myeloid leukemia. J Med Virol.

[28] Phasuk N, Keatkla J, Rattanasiri S, Techasaensiri C, Anurathapan U, Apiwattanakul N (2019). Monitoring of cytomegalovirus infection in non-transplant pediatric acute lymphoblastic leukemia patients during chemotherapy. Medicine (Baltimore).

[29] Balakrishnan K, Adams LE (1995). The role of the lymphocyte in an immune response. Immunol Investig.

[30] Zerr DM, Corey L, Kim HW, Huang ML, Nguy L, Boeckh M (2005). Clinical outcomes of human herpesvirus 6 reactivation after hematopoietic stem cell transplantation. Clin Infect Dis.

[31] Ogata M, Kikuchi H, Satou T, Kawano R, Ikewaki J, Kohno K, Kashima K, Ohtsuka E, Kadota J (2006). Human herpesvirus 6 DNA in plasma after allogeneic stem cell transplantation: incidence and clinical significance. J Infect Dis.

[32] Elmaagacli AH, Steckel NK, Koldehoff M, Hegerfeldt Y, Trenschel R, Ditschkowski M, Christoph S, Gromke T, Kordelas L, Ottinger HD (2011). Early human cytomegalovirus replication after transplantation is associated with a decreased relapse risk: evidence for a putative virus-versus-leukemia effect in acute myeloid leukemia patients. Blood.

[33] Teira P, Battiwalla M, Ramanathan M, Barrett AJ, Ahn KW, Chen M, Green JS, Saad A, Antin JH, Savani BN (2016). Early cytomegalovirus reactivation remains associated with increased transplant-related mortality in the current era: a CIBMTR analysis. Blood.

[34] Litjens NHR, van der Wagen L, Kuball J, Kwekkeboom J (2018). Potential Beneficial Effects of Cytomegalovirus Infection after Transplantation. Front Immunol.

[35] Humar A, Malkan G, Moussa G, Greig P, Levy G, Mazzulli T (2000). Human herpesvirus-6 is associated with cytomegalovirus reactivation in liver transplant recipients. J Infect Dis.

[36] Mendez JC, Dockrell DH, Espy MJ, Smith TF, Wilson JA, Harmsen WS, Ilstrup D, Paya CV (2001). Human beta-herpesvirus interactions in solid organ transplant recipients. J Infect Dis.

[37] Lautenschlager I, Lappalainen M, Linnavuori K, Suni J, Höckerstedt K (2002). CMV infection is usually associated with concurrent HHV-6 and HHV-7 antigenemia in liver transplant patients. J Clin Virol.

[38] Nasimfar A, Sadeghi E, Alborzi A, Sepehrvand N, Ziyaeyan M, Jamalidoust M, Malek-Hosseini SA. The Activation of Cytomegalovirus and Human Herpes Virus 6 After Liver Transplantation. Hepat Mon. 2018;18:e11987.

[39] Abdel Massih RC, Razonable RR (2009). Human herpesvirus 6 infections after liver transplantation. World J Gastroenterol.

[40] Miyoshi H, Tanaka-Taya K, Hara J, Fujisaki H, Matsuda Y, Ohta H, Osugi Y, Okada S, Yamanishi K (2001). Inverse relationship between human herpesvirus-6 and -7 detection after allogeneic and autologous stem cell transplantation. Bone Marrow Transplant.

[41] Boutolleau D, Fernandez C, Andre E, Imbert-Marcille BM, Milpied N, Agut H, Gautheret-Dejean A (2003). Human herpesvirus (HHV)-6 and HHV-7: two closely related viruses with different infection profiles in stem cell transplantation recipients. J Infect Dis.

[42] Kidd IM, Clark DA, Sabin CA, Andrew D, Hassan-Walker AF, Sweny P, Griffiths PD, Emery VC (2000). Prospective study of human betaherpesviruses after renal transplantation: association of human herpesvirus 7 and cytomegalovirus co-infection with cytomegalovirus disease and increased rejection. Transplantation.

[43] Tormo N, Solano C, de la Camara R, Garcia-Noblejas A, Cardenoso L, Clari MA, Nieto J, Lopez J, Hernandez-Boluda JC, Remigia MJ (2010). An assessment of the effect of human herpesvirus-6 replication on active cytomegalovirus infection after allogeneic stem cell transplantation. Biol Blood Marrow Transplant.

[44] Blazquez-Navarro A, Dang-Heine C, Wittenbrink N, Bauer C, Wolk K, Sabat R, Westhoff TH, Sawitzki B, Reinke P, Thomusch O (2018). BKV, CMV, and EBV Interactions and their Effect on Graft Function One Year Post-Renal Transplantation: Results from a Large Multi-Centre Study. EBioMedicine.

[45] Aalto SM, Linnavuori K, Peltola H, Vuori E, Weissbrich B, Schubert J, Hedman L, Hedman K (1998). Immunoreactivation of Epstein-Barr virus due to cytomegalovirus primary infection. J Med Virol.

[46] Razonable RR, Brown RA, Humar A, Covington E, Alecock E, Paya CV, Group PVS (2005). Herpesvirus infections in solid organ transplant patients at high risk of primary cytomegalovirus disease. J Infect Dis.

[47] Wang X, Yang K, Wei C, Huang Y, Zhao D (2010). Coinfection with EBV/CMV and other respiratory agents in children with suspected infectious mononucleosis. Virol J.

[48] Sánchez-Ponce Y, Varela-Fascinetto G, Romo-Vázquez JC, López-Martínez B, Sánchez-Huerta JL, Parra-Ortega I, Fuentes-Pananá EM, Morales-Sánchez A. Simultaneous Detection of Beta and Gamma Human Herpesviruses by Multiplex qPCR Reveals Simple Infection and Coinfection Episodes Increasing Risk for Graft Rejection in Solid Organ Transplantation. Viruses. 2018;10:730. 10.3390/v10120730 PMC6316002 30572622

[49] Schmidt CA, Oettle H, Peng R, Binder T, Wilborn F, Huhn D, Siegert W, Herbst H (2000). Presence of human beta- and gamma-herpes virus DNA in Hodgkin’s disease. Leuk Res.

[50] Wang FZ, Dahl H, Linde A, Brytting M, Ehrnst A, Ljungman P (1996). Lymphotropic herpesviruses in allogeneic bone marrow transplantation. Blood.

[51] Rieger CT, Rieger H, Kolb HJ, Peterson L, Huppmann S, Fiegl M, Ostermann H (2009). Infectious complications after allogeneic stem cell transplantation: incidence in matched-related and matched-unrelated transplant settings. Transpl Infect Dis.

[52] Chapenko S, Krumina A, Logina I, Rasa S, Chistjakovs M, Sultanova A, Viksna L, Murovska M (2012). Association of active human herpesvirus-6, −7 and parvovirus b19 infection with clinical outcomes in patients with myalgic encephalomyelitis/chronic fatigue syndrome. Adv Virol.

[53] Klein SL (2012). Sex influences immune responses to viruses, and efficacy of prophylaxis and therapeutic treatments for viral diseases. BioEssays.

[54] Fernandez-Ruiz M, Lopez-Medrano F, Allende LM, Andres A, Garcia-Reyne A, Lumbreras C, San-Juan R, Morales JM, Paz-Artal E, Aguado JM (2014). Kinetics of peripheral blood lymphocyte subpopulations predicts the occurrence of opportunistic infection after kidney transplantation. Transpl Int.

[55] Calarota SA, Zelini P, De Silvestri A, Chiesa A, Comolli G, Sarchi E, Migotto C, Pellegrini C, Esposito P, Minoli L (2012). Kinetics of T-lymphocyte subsets and posttransplant opportunistic infections in heart and kidney transplant recipients. Transplantation.

[56] Dendle C, Mulley WR, Holdsworth S. Can immune biomarkers predict infections in solid organ transplant recipients? A review of current evidence. Transplant Rev (Orlando). 2019;33:87–98. 10.1016/j.trre.2018.10.001 30551846

[57] Corona-Nakamura AL, Monteon-Ramos FJ, Troyo-Sanroman R, Arias-Merino MJ, Anaya-Prado R. Incidence and predictive factors for cytomegalovirus infection in renal transplant recipients. Transplant Proc. 2009;41:2412–5. 10.1016/j.transproceed.2009.05.008 19715936

[58] Yue C, Kang Z, Ai K, Xu D, Wu J, Pan Y, Yan J, Liu M, Liu Q (2016). Virus infection facilitates the development of severe pneumonia in transplant patients with hematologic malignancies. Oncotarget.

[59] Schots R, Kaufman L, Van Riet I, Ben Othman T, De Waele M, Van Camp B, Demanet C (2003). Proinflammatory cytokines and their role in the development of major transplant-related complications in the early phase after allogeneic bone marrow transplantation. Leukemia.

[60] Nichols WG, Corey L, Gooley T, Davis C, Boeckh M (2002). High risk of death due to bacterial and fungal infection among cytomegalovirus (CMV)-seronegative recipients of stem cell transplants from seropositive donors: evidence for indirect effects of primary CMV infection. J Infect Dis.

[61] Humar A, Kumar D, Caliendo AM, Moussa G, Ashi-Sulaiman A, Levy G, Mazzulli T (2002). Clinical impact of human herpesvirus 6 infection after liver transplantation. Transplantation.

[62] Boeckh M, Leisenring W, Riddell SR, Bowden RA, Huang ML, Myerson D, Stevens-Ayers T, Flowers ME, Cunningham T, Corey L (2003). Late cytomegalovirus disease and mortality in recipients of allogeneic hematopoietic stem cell transplants: importance of viral load and T-cell immunity. Blood.

[63] Hammond SP, Marty FM, Bryar JM, DeAngelo DJ, Baden LR (2010). Invasive fungal disease in patients treated for newly diagnosed acute leukemia. Am J Hematol.

[64] Nguyen Q, Estey E, Raad I, Rolston K, Kantarjian H, Jacobson K, Konoplev S, Ghosh S, Luna M, Tarrand J, Whimbey E (2001). Cytomegalovirus pneumonia in adults with leukemia: an emerging problem. Clin Infect Dis.

