# Supplementary figures and images for: Co-infections of human herpesviruses (CMV, HHV-6, HHV-7 and EBV) in non-transplant acute leukemia patients undergoing chemotherapy
Source: Virol J. 2020 Mar 17;17:37. doi: 10.1186/s12985-020-01302-4 (PMC7079388; doi:10.1186/s12985-020-01302-4)

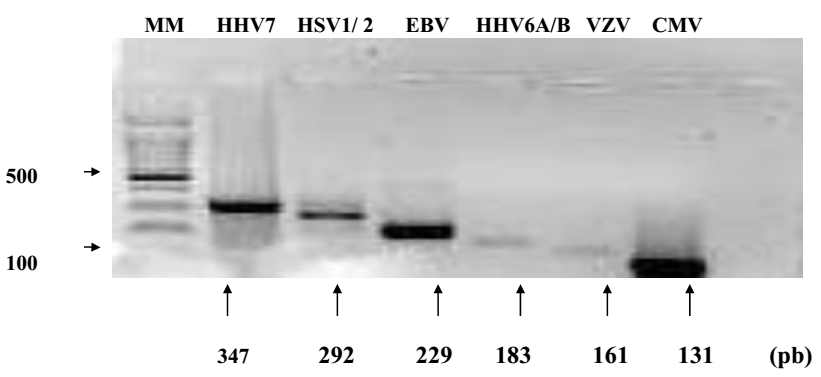


**Figure S1 Specificity of herpesviruses multiplex PCR.**

Supplement: Supplementary file 1 — Additional file 1: Figure S1. Specificity of herpesviruses multiplex PCR. Agarose gel electrophoresis of herpesviruses multiplex PCR showed that a unique PCR product of the expected size was amplified in each positive control. MM: 100pb (base pairs) molecular weight marker and Negative Control (No band). [file 12985_2020_1302_MOESM1_ESM.docx]
